# Do all individuals benefit equally from non‐surgical periodontal therapy? Secondary analyses of systematic review data

**DOI:** 10.1111/jre.13347

**Published:** 2024-09-25

**Authors:** Eero Raittio, Fabio R. M. Leite, Vanessa Machado, João Botelho, Gustavo G. Nascimento

**Affiliations:** ^1^ Department of Dentistry and Oral Health Aarhus University Aarhus Denmark; ^2^ Institute of Dentistry University of Eastern Finland Kuopio Finland; ^3^ National Dental Research Institute Singapore National Dental Centre Singapore Singapore City Singapore; ^4^ Oral Health Academic Clinical Programme Duke‐NUS Medical School Singapore City Singapore; ^5^ Egas Moniz Center for Interdisciplinary Research Egas Moniz School of Health and Science Caparica Portugal

**Keywords:** Bayesian, heterogeneity, non‐surgical periodontal treatment, periodontal diseases, periodontal therapy, periodontitis

## Abstract

**Aims:**

This study aimed to assess the variability and treatment effect heterogeneity in response to non‐surgical periodontal therapy (NSPT).

**Methods:**

Data from randomized controlled trials included in two recent systematic reviews on the effect of NSPT on mean clinical attachment loss (CAL), mean probing pocket depth (PPD), percentage of sites with bleeding on probing (%BOP), PPD ≤3 mm (%PD ≤3 mm), and C‐reactive protein levels (CRP) at 3–12‐month follow‐up among adults with systemic diseases or conditions were used. In these trials, the control arms received no treatment, hygiene advice, or supragingival scaling. The Bayesian meta‐regression models were utilized to assess the variability ratios between NSPT and control groups.

**Results:**

Data from 36 trials on mean PPD, 32 trials on mean CAL, eight trials on %PD ≤3 mm, 31 trials on %BOP and 19 trials on CRP were used. Variability in mean CAL and CRP was approximately 10% higher in the NSPT arms than in the control arms, hinting that there may be room for treatment effect heterogeneity. Instead, variability in mean PPD, %BOP, and %PD ≤3 mm was lower in the NSPT arms than in the control arms.

**Conclusion:**

Potential treatment effect heterogeneity in response to NSPT was observed for CRP and mean CAL. However, substantial measurement error in CAL and natural variation in CRP may contribute to these findings. Conversely, treatment effect heterogeneity appears less pronounced for mean PPD, %BOP, and %PD ≤3 mm, potentially due to greater treatment effects in patients with more severe periodontitis and reduced measurement error in these parameters.

## Graphical abstract

This secondary analysis of systematic review data assessed the variability and treatment effect heterogeneity in response to non‐surgical periodontal therapy.

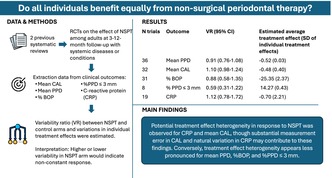

## 1 INTRODUCTION

Non‐surgical periodontal therapy (NSPT) seems to be effective in improving local periodontal health and has been claimed to affect systemic biomarkers.^1^, ^2^, ^3^, ^4^, ^5^ It is commonly assumed that individual responses to periodontal therapy, including NSPT, vary.^4^, ^5^, ^6^ This means there is likely treatment‐by‐patient or treatment‐by‐subgroup interaction in treatment response, reflecting “the extent to which the effects of treatments vary from patient to patient.”^7^ Such an assumption stems from the role played by individual variability in the onset and progression of periodontitis.^6^, ^8^, ^9^, ^10^ Factors such as microbiological and immunological profiles, smoking status, systemic diseases, pregnancy, treatment adherence, and the severity of periodontal disease have been mentioned to influence individual responses to periodontal treatment.^6^, ^11^

Exploring potential treatment effect heterogeneity can be accomplished through several approaches. Optimally, repeated period cross‐over or N‐of‐1 trials, where each trial participant is assigned to treatment and control interventions more than once, would be used.^7^ However, such trial designs in periodontology often appear unfeasible due to challenges like the impossibility of blinding (e.g., in oral hygiene advice), longstanding effects (e.g., if one changes oral hygiene habits “permanently” due to advice given), or the irreversibility of some treatments (e.g., tooth extractions).

In these situations, treatment effect heterogeneity can be explored via treatment‐by‐subgroup interactions in traditional parallel‐group randomized controlled trials (RCTs). The main challenge with traditional a priori specified or post‐hoc subgroup analyses is that most RCTs lack the power to detect meaningful interactions between subgroups. Additionally, information about potentially meaningful subgroups may also be lacking or insufficient, for example, due to unavoidable measurement error.^7^, ^12^

**FIGURE 1:**
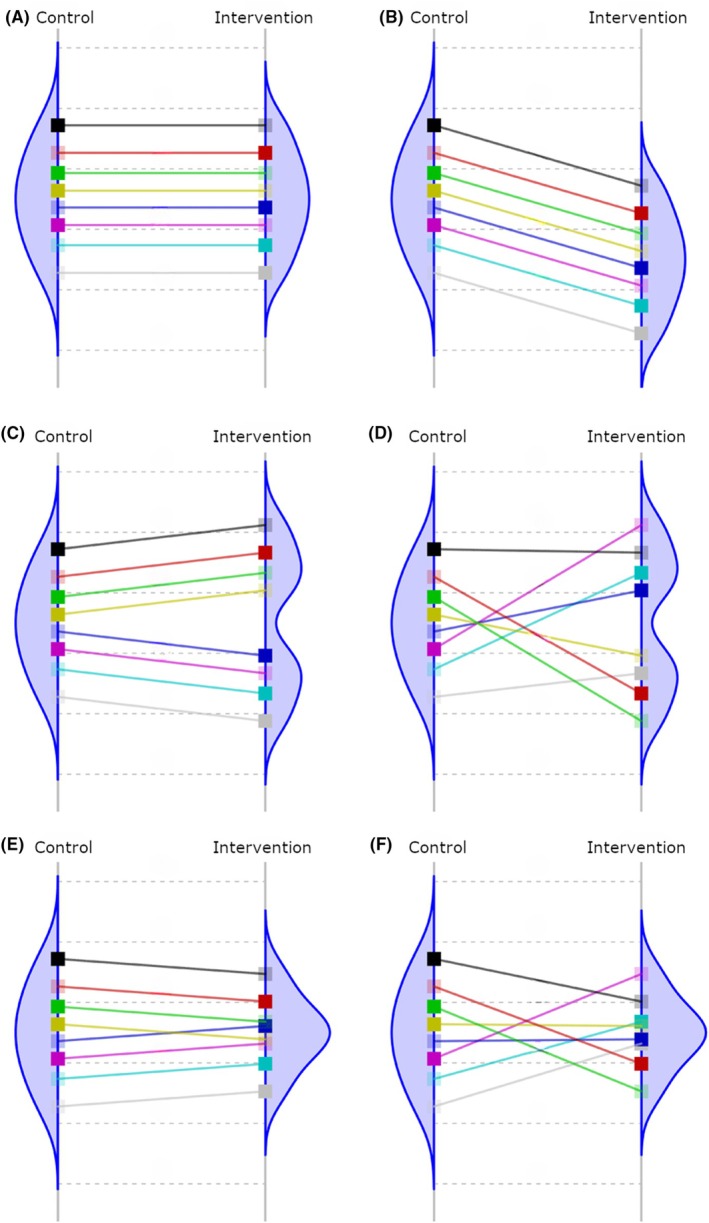
Variability in continuous outcome in control and intervention arms at the end of follow‐up in six hypothetical randomized controlled trials involving eight participants. Adapted from Cortes et al.^13^ Lines between the arms indicate the non‐observable effect for each individual. Densities are the potential distributions of the outcome in each arm. A represents a situation where there is no treatment effect and outcome variability does not change; B represents a situation where treatment effect is constant and outcome variability does not change; C represents a situation where there is treatment‐by‐subgroup interaction, and treatment effect is constant within the two subgroups, outcome variability increases by treatment; D represents a situation where treatment effect varies by each patient and outcome variability increases by treatment; E represents a situation where there is treatment‐by‐subgroup interaction, and treatment effect is constant within the two subgroups, however, outcome variability decreases by treatment; and F represents a situation where treatment effect varies by each patient, and outcome variability decreases by treatment.

One relatively simple way to investigate treatment effect heterogeneity is to analyze variability in outcomes between arms in parallel RCTs at the end of follow‐up following a relatively simple heuristic. If there is no treatment effect or if it is constant, the variance of a continuous outcome would remain constant and similar between arms (Figure 1A,B). However, if there is sufficient strong treatment effect heterogeneity in enough large subpopulation(s) stemming from treatment‐by‐patient or treatment‐by‐subgroup interactions, one could expect an increase (Figure 1C,D) or decrease (Figure 1E,F) in outcome variability between arms over time.

Therefore, the outcome variability in the NSPT arms was compared with the outcome variability in the control arms, focusing on measures of periodontal health and systemic biomarkers. It was hypothesized that the frequently emphasized heterogeneity in the effects of NSPT would be reflected by a clinically meaningful increase or decrease in the outcome variability in the NSPT arm when compared to the control arm. This would support the assumption that there may be treatment effect heterogeneity.^7^, ^13^, ^14^, ^15^, ^16^

## 2 METHODS

### 2.1 Study characteristics and selection

Following a secondary design approach, all primary studies (RCTs) from two recent systematic reviews on the effect of NSPT^1^, ^2^ were selected. These reviews were chosen based on the assumption that systemic diseases or conditions would affect the effect of NSPT, potentially leading to treatment heterogeneity. No updated literature searches were conducted because both reviews were relatively recent.

Luthra et al.^2^ investigated the effect of NSPT, with or without the use of adjunctive therapy, such as antiseptics and/or local antibiotics, on CRP levels among adults with a follow‐up period of 6 months or longer. Although the eligibility criteria for this meta‐analysis did not specify it, most of the primary studies included only people with health conditions (e.g., diabetes and cardiovascular disease) or pregnant.

Another meta‐analysis^1^ investigated the effect of NSPT, with or without the use of adjunctive therapies, on mean clinical attachment loss (CAL), mean probing pocket depth (PPD), the percentage of sites with PD ≤3 mm (%PD ≤3 mm), and the percentage of sites with bleeding on probing (%BOP) among adults with systemic diseases or conditions, such as diabetes, cardiovascular disease, pregnancy, and erectile dysfunction, with follow‐up periods of 3 months or longer.

In both reviews, the control arm received either no treatment, oral hygiene instructions, and supragingival tooth cleaning and polishing, or a combination of the latter two. However, if the control intervention included NSPT, those studies were excluded.

### 2.2 Data extraction

Both meta‐analyses provided relevant data on the primary studies. The following data were used from the primary studies: the number of participants in intervention and control groups, mean of the outcome, and its corresponding standard deviation for intervention and control groups at baseline and at one or two follow‐up time points (CRP at 6 and +12 months; CAL, %PD ≤3 mm and %BOP at 3 or +6 months).

Additionally, information on the risk of bias assessment (high/not high), whether the study included current or former smokers, and the type of control intervention (no intervention, supragingival scaling, and oral hygiene advice) was gathered from the meta‐analysis data. Because one meta‐analysis^1^ did not provide data on current or former smokers, the first author extracted this information from the primary studies.

### 2.3 Data analyses

In line with earlier studies on treatment effect heterogeneity,^15^, ^16^, ^17^, ^18^ a Bayesian linear random‐effects meta‐regression of the natural logarithm of variability ratio (lnVR) between the control (c) and treatment (t) arms was conducted at the end of the follow‐up. The Bayesian meta‐analyses are particularly effective with small sample sizes because they can incorporate prior knowledge and provide posterior estimates that capture uncertainty in the estimated parameters.

Specifically, the natural logarithm of variability ratio, lnVR, is defined as follows:

$$\text{lnVR}=\ln\left(\frac{{\mathrm{SD}}_{t}}{{\mathrm{SD}}_{c}}\right)+\frac{1}{2\left(n_{t}-1\right)}-\frac{1}{2\left(n_{c}-1\right)}$$

where SD stands for standard deviation and *n* stands for number of participants in control (*c*) and treatment (*t*) groups. One can also take the exponent of lnVR so that it can be interpreted as variability ratio (VR), where 1 indicates no difference in variability between the treatment and control arms, <1 indicates higher variance in the control group, and >1 indicates higher variance in the treatment group. For instance, a VR of 2 represents two times higher standard deviation in the treatment group than in the control group, whereas a VR of 0.5 represents half a lower standard deviation.

The standard deviation of the outcome likely depends on the mean of the outcome. This is particularly relevant for this study due to potential “floor” or “ceiling” effects.^16^ For example, measures such as CRP, mean CAL, %BOP, mean PPD, or %PPD ≤3 mm have natural limits (e.g., cannot be below zero or above 100%). Consequently, the group with outcomes closer to these limits will likely have a lower standard deviation compared to the other group. The lnVR was used instead of the coefficient of variation ratio (lnCVR) because lnVR provides greater flexibility in modeling this relationship.^16^ Unlike lnCVR, lnVR does not directly incorporate outcome means into its calculation.

To control for the relationship between the mean and standard deviation, the natural logarithm of the endpoint ratio (lnER) was calculated and included as a covariate in the regression model,^16^ as follows:

$${\text{lnVR}}_{\mathrm{end}}=\alpha +\beta_{1}*\ln\left(\frac{{\bar{x}}_{t}}{{\bar{x}}_{c}}\right)+\beta_{2}*{\text{lnVR}}_{\text{baseline}}=\alpha +\beta_{1}\text{lnER}+\beta_{2}{\text{lnVR}}_{\text{baseline}}$$

where α is an intercept, $\beta_{1}$ is a coefficient capturing the effect of relative differences between the treatment and control arms within each study on variability, and $\bar{x}$ denotes the means of outcome in control and treatment groups at the endpoint. Additionally, the models included random effects to capture between‐study heterogeneity (Tau) and baseline lnVR to account for baseline differences in variability between arms and included random intercepts for each primary study.

Using the Bayesian regression allowed the specification of weakly informative priors (expectations) for model parameters, improving the analysis of small sample sizes. This approach provides some structure to stabilize estimates while still allowing the limited data to influence the results substantially. The use of weakly informative priors resulted in slightly narrower credibility intervals than analyses entirely without. Previous variability ratio analyses in various settings have only indicated modest variability differences between the treatment and control arms,^14^, ^15^, ^16^, ^18^ with lnVRs ranging from −0.2 to 0.2. Therefore, a normal distribution with a mean of 0 and a standard deviation of 0.5 was specified as the prior for the intercept (lnVR). For between‐study variance (Tau) and the coefficient for lnER, Cauchy priors with a location of 0 and a scale of 1 were specified.^15^, ^18^ In sensitivity analyses, the control intervention (no intervention, supragingival scaling, or oral hygiene advice—Model 2), smoking (Model 3), and risk of bias evaluation (Model 4) were included as additional covariates.

Earlier studies have suggested varying responses to periodontal therapy according to the baseline condition severity.^3^, ^19^, ^20^ Therefore, the baseline outcome value in the intervention arm was included in Model 4 as an additional covariate, and its coefficient was interpreted to determine whether the baseline condition severity was associated with outcome variability in the follow‐up.

From lnVR, it is possible to estimate the treatment effect heterogeneity.^16^ Treatment and control groups in RCTs should be exchangeable due to randomization, so what happened in the treatment group is a reasonable estimate of what would have happened in the control group and vice versa. Hence, it can be assumed that the post‐treatment outcome value in an individual is composed of the endpoint outcome value under the control condition plus an additional treatment effect if the individual receives the intervention.^15^, ^16^, ^18^ With the above‐estimated lnVR, the bounds of treatment effect heterogeneity can be computed as follows:

$${\mathrm{SD}}_{\mathrm{tx}}={\mathrm{SD}}_{c}*\left(\sqrt{\left(e^{\text{lnVR}}-1+\rho^{2}\right)}-\rho \right)$$

where SD_tx_ is the standard deviation of individual treatment effects and ${\mathrm{SD}}_{c}$ is the standard deviation of the post‐treatment outcome value in the control group.^16^ The correlation coefficient 𝜌 denotes the correlation between the endpoint outcome value under the control intervention and the treatment effect.^16^ The lower the 𝜌 is, the larger the standard deviation of individual treatment effects. For instance, a negative correlation indicates that a person who had a below‐average treatment effect on CAL would have shown above‐average CAL in the control arm (Figure 1D,F). Conversely, a positive correlation would indicate that patients with lower CAL in the control arm would also imply a lower treatment effect on CRP, leading to relatively low treatment effect heterogeneity for a given variability ratio (Figure 1C,E).

Because the real strength of this correlation is unknown and unobservable, one can simulate results assuming different strengths. The bounds of treatment effect heterogeneity were calculated by assuming correlations with values ranging from −1.0 to 1.0 in steps of 0.2 and using the VR (95% credibility interval, CI) estimated above.^15^, ^18^ Specifically, the standard deviation of individual treatment effects was calculated for each of the eleven 0.2 steps using a random posterior sample of 1000 VRs ($e^{\text{lnVR}}$) from the Bayesian regression model.

Given the absence of thresholds for minimal clinically relevant differences in the investigated outcomes, the mean differences in the outcomes were estimated with random‐effects meta‐analyses to put the treatment effect heterogeneity in perspective relative to the average treatment effect.

Data were handled and analyzed in R (v. 4.4.1). Data and script are shared via GitHub (raittioe/NSPT). The study was not preregistered due to its nature of secondary analyses of systematic review data. PRISMA guidelines were adhered to as much as appropriate for this type of study.

## 3 RESULTS

After excluding two trials where the control protocol included NSPT, this study included data from 36 trials with 46 time points on mean PPD, 32 trials with 40 time points on mean CAL, eight trials with ten time points on %PPD ≤3 mm, 31 trials with 38 time points on %BOP, and 19 trials with 20 time points on CRP outcome (Table 1 and Table S1). All but two studies included people with one or more health conditions: diabetes mellitus (26 trials), cardiovascular diseases (10 trials), pregnancy (6 trials), rheumatoid arthritis (2 trials), erectile dysfunction (2 trials), obesity (1 trial), polycystic ovarian syndrome (1 trial), chronic obstructive pulmonary disease (1 trial), chronic kidney disease (1 trial), and metabolic syndrome (1 trial). Detailed characteristics of these trials are available in the Supplementary and the two reviews.^1^, ^2^

**TABLE 1:** Numbers and characteristics of the included studies.

Outcome	Studies	Patients	Included smokers	Risk of bias	Control intervention	
	Trials/Follow‐up timepoints, *n*/*n*	Treatment/Control, *n*/*n*	Yes, *n* (%)^a^	High, *n* (%)^a^	Oral hygiene advice, *n* (%)^a^	Supragingival, *n* (%)^a^
Mean periodontal pocket depth (PPD)	36/46	1583/1542	28 (64)	8 (17)	15 (33)	5 (11)
Mean clinical attachment loss (CAL)	32/40	1406/1372	24 (63)	6 (15)	13 (32)	5 (12)
% sites with pocket depth ≤3 mm (%PPD ≤3 mm)	8/10	232/216	3 (30)	1 (10)	2 (20)	2 (20)
% sites with bleeding on probing (%BOP)	31/38	1389/1341	26 (70)	4 (11)	8 (21)	9 (24)
C‐reactive protein (CRP)	19/20	1043/988	14 (70)	5 (25)	10 (50)	5 (25)

^a^ *n* (%) of follow‐up timepoints.

Table 2 summarizes the results of the variability analyses. The variability in mean CAL was approximately 10% higher (95% CI: −2%; 24%) in NSPT groups than in control groups. The variability in mean PPD and %BOP was approximately 10% lower in the NSPT arms than in the control arms, while the variability of CRP was approximately 10% higher in the NSPT arms. Variability in the %PPD ≤3 mm was considerably lower (41%) in the NSPT arms, but the estimate presented significant uncertainty due to the low number of studies and between‐study heterogeneity.

**TABLE 2:** Comparison of the variability in the treatment groups with the variability in the control groups across the five outcomes and different adjustment sets. Variability ratio, VR, over (less) than 1 indicates higher variability in the treatment (control) groups. Tau represents between‐study heterogeneity.

Outcome	Model 1 (controls for VR baseline and endpoint ratio)		Model 2: Model 1 + control intervention		Model 3: Model 2 + smoking^a^		Model 4: Model 3 + high risk of bias^a^	
	VR (95% CI)	Tau (95% CI)	VR (95% CI)	Tau (95% CI)	VR (95% CI)	Tau (95% CI)	VR (95% CI)	Tau (95% CI)
Mean periodontal pocket depth (PPD)	0.91 (0.76–1.08)	0.29 (0.20–0.41)	0.89 (0.71–1.09)	0.30 (0.20–0.42)	0.86 (0.61–1.19)	0.31 (0.21–0.44)	0.86 (0.61–1.21)	0.31 (0.21–0.43
Mean clinical attachment loss (CAL)	1.10 (0.98–1.24)	0.20 (0.12–0.29)	1.12 (0.97–1.30)	0.20 (0.13–0.30)	1.13 (0.90–1.42)	0.21 (0.13–0.33)	1.13 (0.90–1.43)	0.21 (0.13–0.32)
% sites with pocket depth ≤3 mm (%PPD ≤3 mm)	0.59 (0.31–1.22)	0.80 (0.42–1.57)	0.70 (0.41–1.47)	0.38 (0.03–1.08)	0.90 (0.33–3.16)	0.46 (0.05–1.30)	0.88 (0.27–4.43)	0.58 (0.03–1.95)
% sites with bleeding on probing (%BOP)	0.88 (0.58–1.35)	0.67 (0.51–0.89)	0.92 (0.53–1.67)	0.69 (0.52–0.94)	1.14 (0.56–2.34)	0.64 (0.47–0.88)	1.14 (0.55–2.41)	0.65 (0.48–0.90)
C‐reactive protein (CRP)	1.12 (0.78–1.72)	0.63 (0.41–0.97)	0.96 (0.48–2.15)	0.68 (0.44–1.10)	0.98 (0.40–2.47)	0.72 (0.45–1.16)	0.98 (0.40–2.47)	0.72 (0.45–1.16)

^a^ Two primary studies did not include smoking information, so the number of trials (and observations) were 34 (44) for PD and 30 (38) for CAL for models including smoking.

There was also considerable between‐study heterogeneity in all other outcomes, which was greatest in %BOP, %PPD ≤3 mm, and CRP (Table 2). Adjusting for control intervention (no treatment, oral hygiene advice, and supragingival scaling and root planning), smoking, or risk of bias did not considerably change VR estimates for mean CAL or mean PPD. The adjustments decreased VR estimates for CRP and increased VR for %BOP and %PPD ≤3 mm but also widened the 95% CIs considerably due to small sample sizes and heterogeneity.

A one‐unit higher baseline outcome value in the NSPT arm was associated higher variability in mean PPD (1.17, 95% CI: 1.02–1.35) or %BOP (1.02, 95% CI: 1.00–1.03), but not with CRP (0.77, 95% CI: 0.52–1.11), %PPD ≤3 (0.98, 95% CI: 0.92–1.02), or mean CAL (0.96, 95% CI: 0.89–1.04).

**FIGURE 2:**
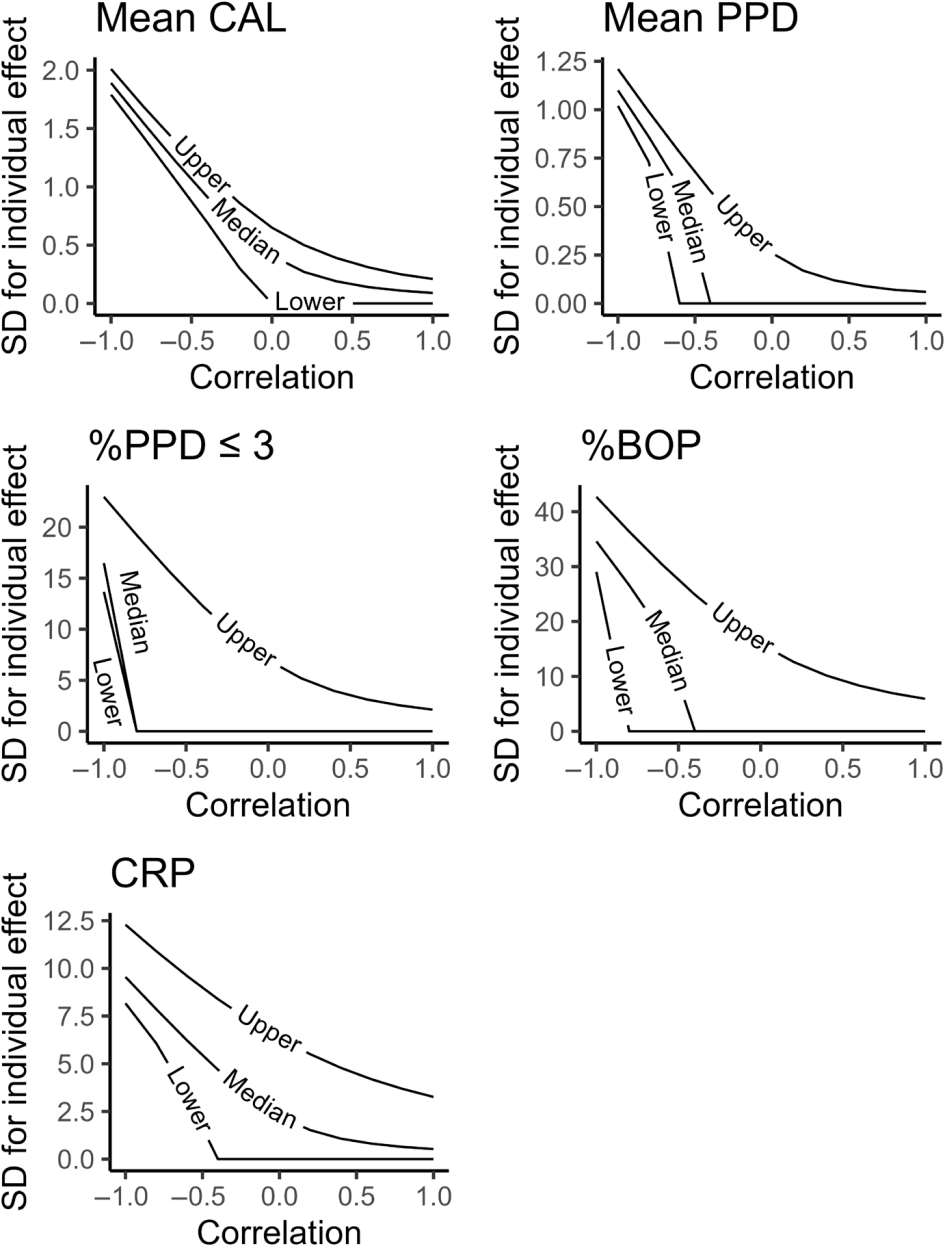
Estimates of the standard deviation of individual treatment effects for the five outcomes assuming different strenghts of correlation between the endpoint outcome value under control intervention and the treatment effect. Lower and upper lines represent 2.5th and 97.5th percentiles, respectively.

**FIGURE 3:**
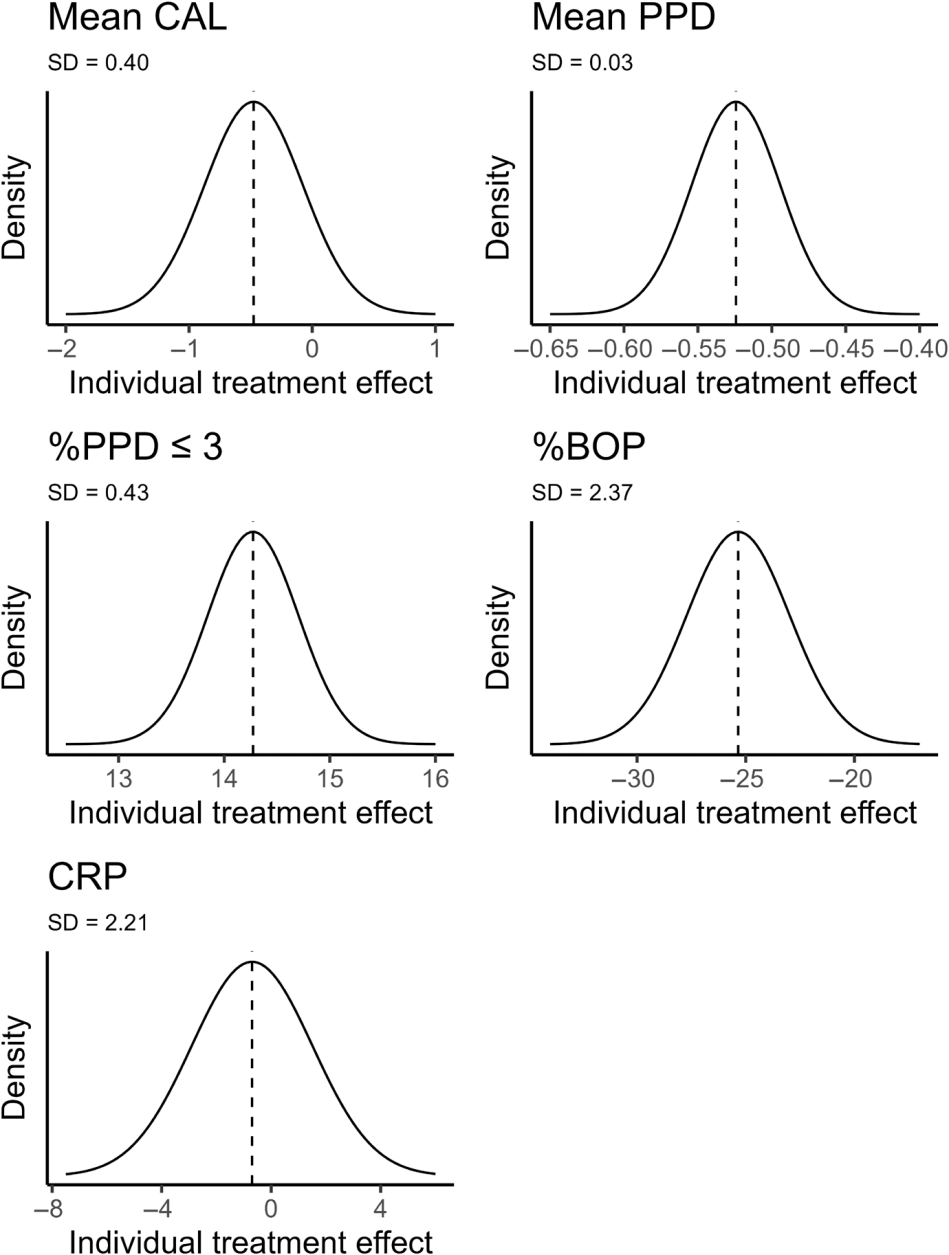
Distribution of individual treatment effects for the five outcomes. Vertical lines represent the estimated average treatment effect. The average estimated standard deviation of individual treatment effects was calculated assuming zero correlation between the endpoint outcome value under the control intervention and the treatment effect.

Table 3 and Figure 2 summarize the estimates of standard deviations of the individual treatment effect based on estimates from Model 1. This analysis shows that if there is no correlation between the treatment effect and the endpoint outcome value under the control condition (the correlation is 0), the SDs for individual treatment effects are 0.00 (median, 2.5th percentile and 97.5th percentile, P2.5‐P97.5: 0.00–0.26) for mean PPD, 0.41 (0.00–0.65) for mean CAL, 0.00 (0.00–6.98) for %PPD ≤3 mm, 0.00 (0.00–15.90) for %BOP, and 2.24 (0.00–6.33) for CRP. Even though there is considerable uncertainty in these estimates, it seems that there is the most room for treatment effect heterogeneity in mean CAL and CRP when comparing the estimated SDs and P2.5‐P97.5 intervals for individual treatment effects to the estimated mean difference between the intervention and control arms (Table 3, Figure 3). As Figure 3 indicates, in mean CAL and CRP, it is relatively likely that there is a considerable proportion of people whose mean CAL or CRP gets worse after the treatment, whereas in mean PPD, %BOP, and %PPD ≤3 mm, practically all do better after treatment. However, depending on the actual variability ratio and the correlation between the treatment effect and the endpoint outcome value under the control condition (Table 3), treatment effect heterogeneity can be much lower or higher than what the assumption of zero correlation indicates (Figure 3).

**TABLE 3:** Estimates of the standard deviation of individual treatment effects for the five outcomes assuming different strengths of correlation between the endpoint outcome value under control intervention and the treatment effect.

Correlation	Mean periodontal pocket depth	Mean clinical attachment loss	% sites with pocket depth ≤3 mm	% sites with bleeding on probing	C‐reactive protein
	Median SD (P2.5‐P97.5)	Median SD (P2.5‐P97.5)	Median SD (P2.5‐P97.5)	Median SD (P2.5‐P97.5)	Median SD (P2.5‐P97.5)
−1	1.10 (1.02–1.21)	1.89 (1.79–2.01)	16.49 (13.67–22.97)	34.61 (29.04–42.07)	9.55 (8.17–12.29)
−0.8	0.86 (0.73–0.99)	1.55 (1.43–1.69)	0.00 (0.00–19.22)	26.60 (0.00–36.37)	7.86 (6.07–10.90)
−0.6	0.60 (0.00–0.78)	1.22 (1.06–1.39)	0.00 (0.00–15.63)	17.86 (0.00–30.38)	6.22 (3.30–9.6)
−0.4	0.00 (0.00–0.58)	0.91 (0.05–1.11)	0.00 (0.00–12.30)	0.00 (0.00–24.87)	4.69 (0.00–8.39)
−0.2	0.00 (0.00–0.40)	0.63 (0.02–0.86)	0.00 (0.00–9.37)	0.00 (0.00–20.00)	3.32 (0.00–7.3)
0	0.00 (0.00–0.26)	0.41 (0.00–0.65)	0.00 (0.00–6.98)	0.00 (0.00–15.9)	2.24 (0.00–6.33)
0.2	0.00 (0.00–0.17)	0.27 (0.00–0.50)	0.00 (0.00–5.20)	0.00 (0.00–12.64)	1.52 (0.00–5.50)
0.4	0.00 (0.00–0.12)	0.19 (0.00–0.39)	0.00 (0.00–3.96)	0.00 (0.00–10.16)	1.07 (0.00–4.78)
0.6	0.00 (0.00–0.09)	0.14 (0.00–0.31)	0.00 (0.00–3.12)	0.00 (0.00–8.32)	0.81 (0.00–4.18)
0.8	0.00 (0.00–0.07)	0.11 (0.00–0.25)	0.00 (0.00–2.54)	0.00 (0.00–6.95)	0.64 (0.00–3.68)
1	0.00 (0.00–0.06)	0.09 (0.00–0.21)	0.00 (0.00–2.12)	0.00 (0.00–5.92)	0.53 (0.00–3.26)
Estimated average treatment effect, mean difference (95% CI)	−0.52 (−0.63; −0.42)	−0.48 (−0.59; −0.36)	14.27 (6.84; 21.71)	−25.35 (−29.66; −21.03)	−0.70 (−1.04; −0.36)
Variability ratio, VR (95% CI)	0.91 (0.76–1.08)	1.10 (0.98–1.24)	0.59 (0.31–1.22)	0.88 (0.58–1.35)	1.12 (0.78–1.72)

P2.5‐P97.5: 2.5th percentile and 97.5th percentile.

## 4 DISCUSSION

### 4.1 Main findings

These secondary analyses of systematic review data revealed differential variability in outcomes between the NSPT and control arms across various measures. Mean CAL showed slightly increased variability in the NSPT arms compared to the control arm after 3–6‐month follow‐ups. Similarly, variability in CRP was slightly higher in the NSPT arms than in the control arms. Conversely, variability in mean PPD, %BOP, and %PPD ≤3 mm was lower in the NSPT arms than in the control arms after a 3–6‐month follow‐up. These findings suggest potential treatment effect heterogeneity in mean CAL and CRP in relation to the expected average treatment effect. The higher (worse) baseline outcome value was associated with higher variability at the follow‐up in terms of mean PPD and %BOP.

### 4.2 Limitations

The represented treatment effect heterogeneity estimates are very uncertain because the authors did not have information about the actual correlation between control response and treatment effect or data sharing of already conducted studies on the topic. This warrants further research. One way to investigate the strength of these correlations is to record periodontal status before and after the few months of the oral hygiene phase,^21^, ^22^ including oral hygiene instructions and supragingival scaling, and then again after the NSPT. This would help estimate treatment effect heterogeneity much more reliably than in this study.

Like in all systematic reviews and meta‐analyses, findings are bound by the limitations of primary studies. The investigated outcomes are surrogate endpoints (not necessarily patient‐important ones) over 3–12 months. Therefore, it is unclear whether these findings can effectively inform clinical decision‐making for patients or clinicians; CRP, mean CAL, or mean PPD is not commonly used in clinical decision‐making. Mean CAL and mean PPD likely overlook relevant variations in CAL and PPD across individual sites. In addition, the relatively low number of primary studies, coupled with considerable heterogeneity between them in terms of sample attrition, follow‐up period, measurements, and reporting (e.g., intention‐to‐treat/per‐protocol and median/mean or rounding), presents a challenge.^1^, ^2^ For instance, it is problematic that some studies reported only means and standard deviations for per‐protocol design for baseline and follow‐up, as the numbers are not protected against confounding by randomization.

Furthermore, different control interventions and NSPT protocols can also introduce considerable between‐study heterogeneity,^1^, ^2^ which cannot be completely controlled for. For instance, the combination of oral health instructions and supragingival scaling seems to have a stronger effect on periodontal tissues compared to no treatment protocol in periodontitis patients,^23^ whereas the effect of NSPT, and, thus, variability in outcomes, may be affected by whether adjunctive therapies, such as antiseptics and/or local and/or systemic antibiotics, were used.^24^ Moreover, some of the included primary studies had considerable baseline variability differences between arms, implying possible problems with random allocation, which is not only worrisome but may also be due to the normality assumption behind variability measures. As an example, in the study by Kolte et al.,^25^ there were 2.05 times (95% CI: 1.43–2.95) higher variability in mean CAL and 1.81 (95% CI: 1.26–2.60) higher variability in mean PPD in the intervention arm than in the control arm already at the baseline. Additionally, the investigated studies included almost only patients with systemic diseases or conditions,^1^, ^2^ which may be associated with stronger and more common treatment‐by‐patient/subgroup interactions than one would see in more general periodontitis populations. This may have inflated the estimates of the effect of treatment on heterogeneity found in this study.

Finally, the short‐term effects of the studies limited the evaluation to parameters such as BOP, CAL, and PPD, which are heterogeneous indicators of periodontal progression.^26^ Future studies examining the variability in NSPT should prioritize objective and patient‐important endpoints, such as tooth loss, which would require longer follow‐up periods and be more relevant from the patient's perspective.^27^, ^28^

### 4.3 Interpretation of findings

Treatment effect heterogeneity in periodontal parameters can be attributed to various factors, including environmental, microbial, genetic, and immunological factors, with smoking likely being the strongest contributor.^2^, ^6^, ^10^, ^29^ The baseline severity of periodontitis offers another plausible explanation for differing outcome variability. Sites with higher initial PPD show greater PPD reduction,^3^, ^19^, ^20^ resulting in lower variability in outcomes such as mean PPD, %BOP, and %PPD ≤3 mm in the NSPT arms than in the control arms (Figure 1E,F). In line with these findings, it was found that in terms of mean PPD and %BOP, the more severe the baseline condition at the commencement of the trial, the higher the variability difference between the arms at the follow‐up. Additionally, reduced measurement error in the NSPT arms may contribute to lower variability in PPD measures, as shallower pockets are less prone to measurement error than deeper ones.^30^, ^31^

The slightly increased variability and treatment effect heterogeneity in mean CAL and CRP after NSPT, despite following similar principles of greater benefit in worse conditions, warrants further exploration. Several factors may contribute to this observation. Treatment effect heterogeneity from known or unknown sources may be stronger in mean CAL than in other outcomes. The stability of CAL after treatment also differs from more responsive inflammatory parameters (e.g., PPD and BOP). For instance, sites with initial shallow periodontal pockets may show attachment loss after NSPT.^19^, ^32^ Accordingly, it was found that worse baseline mean CAL was not significantly associated with higher or lower variability in the follow‐up. This could potentially inflate variability in the NSPT arms (as illustrated by the red line in Figure 1D), particularly in studies including only individuals with very mild conditions at baseline, such as those including only pregnant women. Additionally, clinically relevant changes in CAL typically require a longer observation period. Furthermore, CAL measurement is more susceptible to measurement error, particularly if one measures CAL indirectly by summing PPD and gingival recession.^33^ The slightly higher CAL variability observed in the NSPT groups could thus stem from a decrease in the sources of CAL measurement error, such as the elimination of subgingival calculus, deep pockets, and bleeding.^33^ Lastly, potential treatment effect heterogeneity may arise from differences between factors related to the inflammatory nature of periodontitis and those driving the destructive aspect of the disease.^34^

There likely exist several environmental, microbial, genetic, inflammatory, and immunological factors, such as obesity,^35^ which contribute to slightly increased variability in CRP and potential treatment effect heterogeneity of NSPT.^2^, ^36^ However, at least, there is considerably high intra‐individual variation in CRP levels between weeks and months.^37^, ^38^ As an example, it has been shown that “an individual with a CRP measurement precisely at the high‐risk cutoff of 2 mg/L may be expected on repeated sampling [over months] to have measurements that would lie between 0.74 and 3.26 mg/L (2 mg/L ±2 SD).”^38^ This inherent variability in CRP levels underscores the complexity of interpreting the findings from the studies, typically with two to three CRP measures over 6–12 months. While NSPT aims to reduce local periodontal inflammation, its impact on CRP may be masked or modulated by this natural fluctuation, necessitating careful interpretation of study results and potentially multiple measurements over time to accurately assess treatment efficacy and treatment effect heterogeneity on systemic inflammatory markers.

## 5 CONCLUSIONS

### 5.1 Implications for research

There are some implications for further research. Firstly, enhancing the quality of reporting and prioritizing outcomes that matter most to patients are critical steps forward. This study aimed to inspire further investigations into treatment effect heterogeneity within the context of systematic reviews. Beyond mere subgroup analyses, robust methodologies exist for assessing treatment effect heterogeneity in RCTs.^39^, ^40^ Notably, these methods require information on potential subgroups and adequate power for such analyses. It also merits recognition that the traditional but flawed responder analysis—categorizing patients (or sites) as responders or non‐responders based on an arbitrary threshold—is discouraged.^41^, ^42^, ^43^ Overall, there is a need for rigorous research on treatment effect heterogeneity to help refine periodontitis classification and risk assessment practices. These frameworks currently presuppose treatment effect heterogeneity but lack solid empirical support.^28^, ^44^

### 5.2 Implications for clinical practice

Potential treatment effect heterogeneity in response to NSPT was observed for CRP and mean CAL. However, substantial measurement error in CAL and natural variation in CRP may contribute to these findings. Conversely, treatment effect heterogeneity appears less pronounced for mean PPD, %BOP, and %PPD ≤3 mm, potentially due to greater treatment effects in patients with more severe periodontitis and reduced measurement error in these parameters.

In other words, a clinician can expect their patients to become more similar on average in terms of mean PPD, %BOP, and %PPD ≤3 mm after NSPT, whereas, in terms of CRP and mean CAL, they may be slightly more different on average after the treatment. Whether these indicate that there are individual treatment effects that could be harnessed by personalized NSPT remains an open question.

## FUNDING INFORMATION

The study did not receive external funding.

## CONFLICT OF INTEREST STATEMENT

The authors declare that they have no conflict of interest with respect to the publication of this article.

## Supporting information


Table S1:

## Data Availability

The data and analysis script that support the findings of this study are openly available on GitHub at https://github.com/raittioe/NSPT.
